# Authentication of Covid-19 Vaccines Using Synchronous Fluorescence Spectroscopy

**DOI:** 10.1007/s10895-022-03136-5

**Published:** 2023-01-07

**Authors:** Sulaf Assi, Ismail Abbas, Basel Arafat, Kieran Evans, Dhiya Al-Jumeily

**Affiliations:** 1 Pharmacy and Biomolecular Sciences, Liverpool John Moores University, James Parson Tower, Liverpool, L3 3AF UK; 2 Faculty of Science, Lebanese University, Beirut, Lebanon; 3 Faculty of Health, Education, Medicine and Social Care, Bishops Hall Lane, Chelmsford, CM1 1SQ UK; 4 Perkin Elmer, Chalfont Road, Seer Green, Buckinghamshire, HP9 2FX UK; 5 School of Computer Sciences, Liverpool John Moores University, James Parson Tower, Liverpool, L3 3AF UK

**Keywords:** Covid-19, Vaccines, Synchronous fluorescence, Principal component analysis, Gaussian mixture models, Self organising maps

## Abstract

The present study demonstrates the potential of synchronous fluorescence spectroscopy and multivariate data analysis for authentication of COVID-19 vaccines from various manufacturers. Synchronous scanning fluorescence spectra were recorded for DNA-based and mRNA-based vaccines obtained through the NHS Central Liverpool Primary Care Network. Fluorescence spectra of DNA and DNA-based vaccines as well as RNA and RNA-based vaccines were identical to one another. The application of principal component analysis (PCA), PCA-Gaussian Mixture Models (PCA-GMM)) and Self-Organising Maps (SOM) methods to the fluorescence spectra of vaccines is discussed. The PCA is applied to extract the characteristic variables of fluorescence spectra by analysing the major attributes. The results indicated that the first three principal components (PCs) can account for 99.5% of the total variance in the data. The PC scores plot showed two distinct clusters corresponding to the DNA-based vaccines and mRNA-based vaccines respectively. PCA-GMM clustering complemented the PCA clusters by further classifying the mRNA-based vaccines and the GMM clusters revealed three mRNA-based vaccines that were not clustered with the other vaccines. SOM complemented both PCA and PCA-GMM and proved effective with multivariate data without the need for dimensions reduction. The findings showed that fluorescence spectroscopy combined with machine learning algorithms (PCA, PCA-GMM and SOM) is a useful technique for vaccination verification and has the benefits of simplicity, speed and reliability.

## Background

Medicine counterfeiting is a patient safety concern and public health which consequences range from ineffectiveness of treatment, resistance to treatment and/or lethal effects [1]. Counterfeit medicines can be encountered anywhere across the global supply chain, with any medicine and of any formulation [1, 2]. Whereas no class of medicines is exempt, highly sold medicinal products have higher chance of being counterfeited. Covid-19 vaccines are in high demand globally due to their effectiveness in controlling the Covid-19 pandemic [3]. The demand of Covid-19 vaccines outweighs the available global manufacturing capacity and supply and that introduces a challenge in their availability especially in low and middle income countries [4, 5].

In this respect, the black market for Covid-19 vaccines had been estimated at 400% and attributed mainly to the lock down situations [6]. Counterfeit vaccines of different brands have been reported in many countries including China [7], Honduras [8], India [9], Mexico [8, 10], Poland [8, 10] and South Africa [11]. This urges the need for development of rapid methods for authentication of Covid-19 vaccines wherever they were encountered.

Detection of DNA or mRNA in vaccines is important step in authentication of vaccines. Among methods of detection, spectroscopic methods are so popular due to their being rapid, non-destructive and not requiring extensive sample preparation. Fluorescence spectroscopy offers highly sensitivity and specificity for detection of organic compounds. Synchronous fluorescence (SF), first discovered by Lloyds in 1971, is one of the advanced fluorescence methods where both the excitation (λ_exc_) and emission (λ_emission_) wavelengths are scanned simultaneously such that the wavelength interval (Δλ = λ_emiss_ – λ_exc_) between them is kept constant [12]. Once this wavelength interval is optimised, fluorescence spectra will show better improved resolution, resolvable spectral features, narrower bands and less overlapping spectral components than those encountered with conventional fluorescence. Hence, SF spectroscopy enhances the selectivity of detection of compounds in a mixture alongside maintaining sensitivity.

SF spectroscopy has been popular for characterising compounds in solution form [13–17]. In addition, SF spectroscopy has been used for DNA characterisation [13] where it has shown to be a more specific technique for detecting nucleic acids unlike conventional fluorescence spectroscopy. It is noteworthy to mention that conventional fluorescence of nucleic acids is usually weak and requires the use of fluorescent labels in order increase their fluorescent activity [18]. However, SF occurs in femtoseconds before nucleic acids decay (picoseconds) [19–22]. This detection is attributed to the interaction between nucleobases of DNA that changes the nature of the excited state [23]. SF spectroscopy have also been used for other biomolecules [24–27] due to the sharp emission bands the technique yields and that allows specific characterisation of biological compounds. The narrow spectral bands also allow to characterise compounds in mixtures. Hence, SF spectra offer a fingerprint of the sample that contains multiple ingredients.

Covid-19 vaccines, whether based on DNA or mRNA, contain multiple ingredients including lipid nano-particles and diversity of excipients. When in liquid form, SF would offer an ideal technique for characterising these vaccines non-destructively by providing a spectroscopic signature specific to each vaccine. When combined with machine learning algorithms (MLAs), spectroscopic data of medicines have informed about identity, authenticity, manufacturing sources and/or geographical location [28]. Part of MLAs, artificial neural network (ANN) algorithms have shown accuracy in detecting Covid-19 disease diagnosis and prediction of mortality [29–31] Subsequently, this research utilises SF and MLAs (including ANN) for authentication of DNA- and RNA-based vaccines obtained from different manufacturers. The work explores three MLAs for classification of the measured vaccines and understanding patterns between vaccines of the same and different manufactures.

## Materials and Methods

### Samples

All materials used were of analytical grade. Commercially prepared salmon sperm DNA was obtained from Sigma and suspended in normal saline solution then diluted down prior to measurement. RNA was extracted from HaCaT keratinocytes and diluted with normal saline solution. DNA-based (n = 21) and mRNA-based (n = 21) vaccines were obtained through the NHS Central Liverpool Primary Care Network. In this case, the vaccines obtained were left over vials after six doses had been given to patients. Hence, each vial contained less than 0.5 mL of solution and was further diluted with normal saline prior to measurement. Before the experiment all solutions were stored and maintained at a temperature of 4 °C.

### Instrumentation

**Fig. 1:**
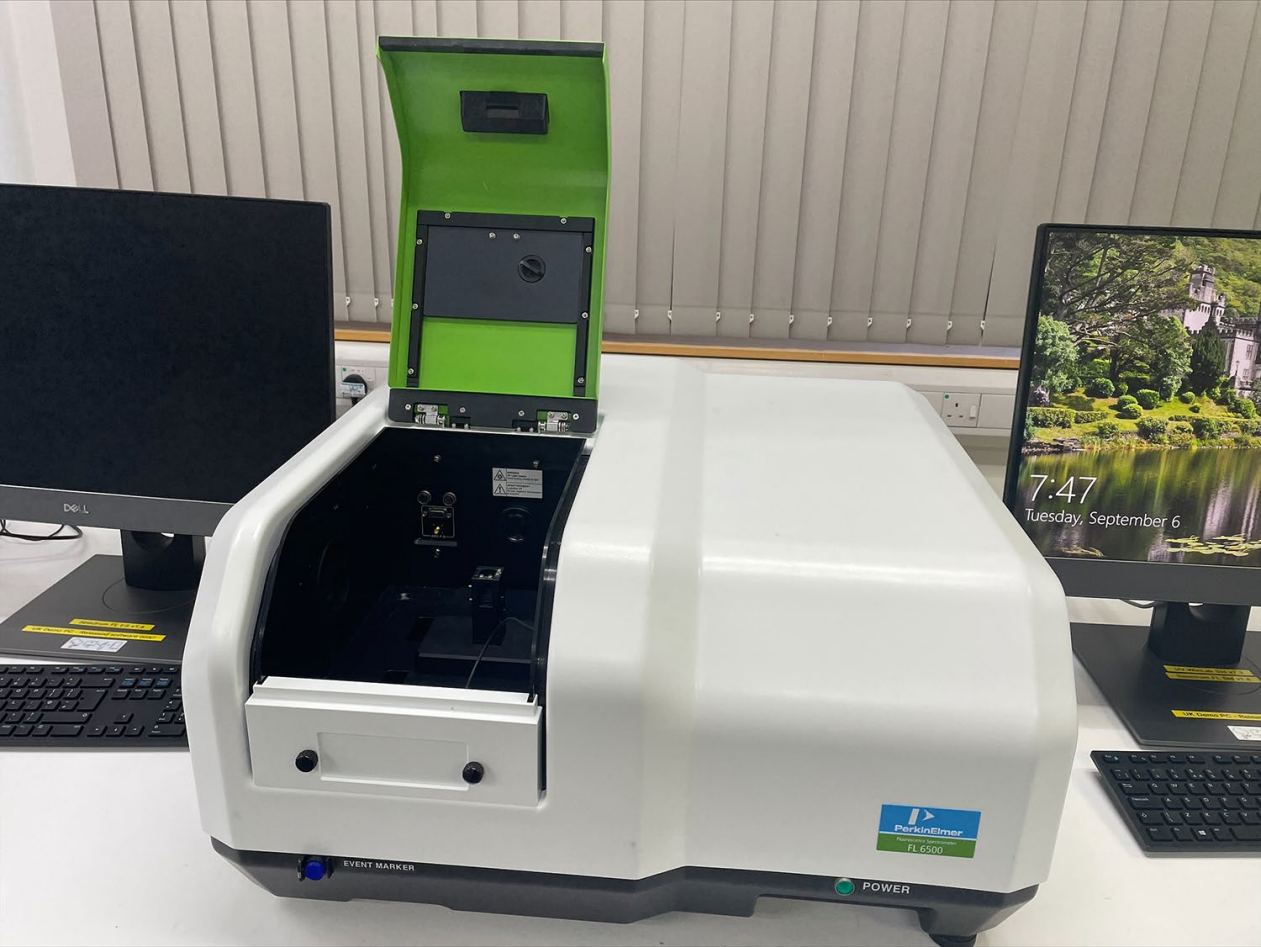
The PerkinElmer FL6500 equipped with a pulsed xenon lamp

2D synchronous spectra were collected using the FL6500 equipped with a pulsed xenon lamp as light source (Fig. 1). Scanning was made over the full range of 230 -700 nm with 30 nm constant difference between excitation and emission wavelengths.

### Procedure

Fluorescence measurements for DNA, RNA and vaccines solutions were obtained after dilution with normal saline solution. Hence, minimal sample intervention was sought in order to keep the sample type as realistic as it could be in a real-world scenario. All synchronous scans were taken using the FL6500 single cell accessory and 10 × 10 mm quartz fluorescence cuvette. The wavelength difference (delta lambda) was 30 nm. Each datapoint was the average of two scans. Background correction was made using normal saline being the solvent in the vaccine samples.

### Data Analysis

Spectral data was imported into Matlab 2019a where spectral visualisation and unsupervised clustering were applied. Unsupervised clustering was undertaken using three different algorithms being: principal component analysis (PCA), PCA-Gaussian Mixture Models (PCA-GMM)) and Self-Organising Maps (SOM) were applied. In all the aforementioned algorithms, the patterns among the different vaccines were observed.

#### Feature Selection

In any data analysis, feature selection is important in terms of what data play a key role in providing meaningful information about the dataset [29]. In this particular study, the data obtained represented spectra that were fingerprint of the measured vaccines. These spectra resulted from the interaction of light with the vaccines and hence represented a fingerprint of the vaccine. Hence, data point on the spectrum was important and played a significant in the identification of the vaccine. Therefore, the full SF spectra were considered when MLAs were applied.

#### PCA

PCA clustered fluorescence spectra according to variances by reducing the dimensions of the spectra into two space, being scores and loading [32]. The PC scores showed the vaccine clusters in multidimensional space whereas the loading showed the significant fluorescence intensities within the PCA model. The relationship between the spectra, scores and loadings is described in Eq. (1):

1 $$X = T. P + Q$$

where,

$X$ is the original data matrix;

$T$ represents the scores;

$P$ represents the loadings;

$Q$ represents the residuals.

Accuracy of the PCA model was evaluated by exploring the grouping among the clusters. Thus, cluster of DNA-based vaccines were expected to be grouped together and separated from other vaccines and vice versa.

#### PCA-GMM

GMM is a probabilistic model that evaluates the distance between the points in an n-dimensional space in a such a way that each dimension is formed by a distinct observation [33]. GM encompasses several Gaussians such that each is identified by k ∈ {1,…, K}, such that K is the number of clusters in the dataset. The multivariate Gaussian distribution has the following three parameters: A mean μ_j_ corresponding to its centre, a covariance Σ_j_ defining its width and a probability $\pi$ defining the size of the Gaussian function. The multivariate Gaussian distribution is explained by Eq. (2):

2 $$P(y) =\sum\nolimits_{j = 1}^{k}{\pi }_{j}f({y}_{i}|{\mu }_{j},{\sum\nolimits}_{j})= \sum\nolimits_{j = 1}^{k}{\phi }_{j}{P}_{j}(y)$$

where,

${\phi }_{j}$ is the mixing proportion for cluster j

The clusters are calculated fitting the maximum likelihood GMM as a function of the set of parameters in Eq. (3):

3 $$\theta = \{{{\phi }_{j}, {\mu }_{j}, {\Sigma }_{j}\}}_{j}^{k} = 1$$

In this respect, GMM was applied to the PCA scores and the accuracy of the PCA_GMM mode was evaluated by the width of the covariance matrix.

#### SOM

SOM offers additional unsupervised clustering approach to PCA and PCA-GMM. SOM has the advantage in the ability to deal with non-linear data and shows them in lower dimensions [34, 35]. Similar to PCA, SOM is able to detect features in the spectra without previously knowing classes or membership about the spectra. Being a neural network mode, SOM consists of organised neurons that can vary from few and up to thousands of neurons [34]. The output of SOM comprises a similarity map of the input data but of lower dimensions. Mapping in SOM is similar to a classical vector quantisation (Eq. (4)):

4 $$X = {\left\{{\varepsilon }_{1}, {\varepsilon }_{2},\dots ,{\varepsilon }_{j}\right\}}^{T }\in {\mathbb{R}}^{n}$$

where,

$X$ is the input vector that is real

ε are the input variables (in this case fluorescence intensity)

j is the number of variables

${\mathbb{R}}$ is a set of real numbers

At the beginning of learning, a parametric real vector (weight) is assigned to each input variable as per Eq. (5):

5 $${w}_{i}= {\left[{w}_{i1},{w}_{2},\dots ,{w}_{in}\right]}^{T} \in {\mathbb{R}}^{n}$$

where,

${w}_{i}$ Is the parametric real vector linked to neuron I on the grid

n is the total number of neurons

If the distance between x and m is d(x, ${m}_{i}$), then the image of the input vector is defined by the array element c that best matches x (Eq. (6)) such that:

6 $$c={\mathrm{arg\;min}}_{i}\{d\left(x, {w}_{i}\right)\}$$

In this respect, c is optimised such that w is close to x and the weight of the winning neuron and its neighbour is updated by Eq. (7) until the map converges:

$$v \left(t\right)=\mathrm{arg}\;{min}_{k \epsilon\Omega } \parallel \varkappa (t) - {w}_{k}(t)\parallel$$

7 $$\Delta {w}_{k}\left(t\right)= \propto \left(t\right)n(v, k, t)[x\left(t\right)- {w}_{v}\left(t\right)]$$

where,

$n (v, k, t)$ is the neighbouring function

$x\left(t\right)$ is the input at time $t$

${w}_{v}\left(t\right)]$ is the weight of the winning neuron at time $t$

## Results and Discussion

This study represented the first application of SF spectroscopy to Covid-19 vaccines. SF showed many advantages relating to the high scanning speed (rapidity) and ability to detect the analyte the DNA and RNA in a sample in the presence of other impurities. SF yielded sharp bands and that made it a robust technique for determining Covid-19 vaccines. SF spectra showed a fingerprint of each type of vaccine that corresponded to the excitation-emission spectra of the vaccines. Moreover, when combined with MLAs, SF was able to differentiate between DNA- and RNA-based vaccines with no prior knowledge of the samples.

### Vaccines Characterisation

**Fig. 2:**
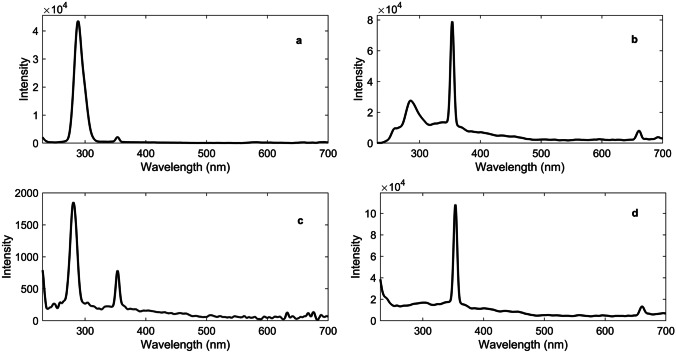
Synchronous fluorescence spectra of **a** DNA, **b** DNA-based vaccine, **c** RNA and **d** RNA-based vaccine solutions diluted in normal saline

The fluorescence spectra of DNA, RNA, DNA-based vaccines and RNA-based vaccines are shown in Fig. 2. The results showed a similarity in between fluorescence spectra of DNA and DNA-based vaccines, and between RNA and RNA-based vaccines. In this respect, DNA showed bands at 288.5 (strong) and 353 (weak) nm that were strong and weak bands and that corresponded to the excitation and emission of thymine (6–4) photoproduct respectively [36]. The (6–4) photoproduct have shown to be a much stronger emitter than DNA itself [37]. This agreed with the previous literature that reported thymine (5–4) photoproduct illumination around 369 nm win synchronous fluorescence spectra when using a delta lambda of 50 nm [38]. Our study has used delta lambda of 10 nm and this could have attributed to the difference in the emission wavelength between both studies. On the other hand, thymine dimer could not be detected and this could be attributed to the lack of conjugation in the dimer in contrary to the photo-product [38]. Likewise, RNA showed key bands at 280.8 and 352.6 nm that related to the uracil (6–4) adduct excitation and emission wavelengths respectively [39]. Nonetheless, vaccine spectra varied slightly from the fluorescence spectra of DNA and RNA by the presence of a band at 660.7 nm and that could be linked to the common excipients in both vaccines. Apart from this band, DNA-based vaccines and mRNA-based vaccines showed corresponding bands to DNA and RNA respectively.

### Classification of Vaccines

#### Principal Component Analysis

**Fig. 3:**
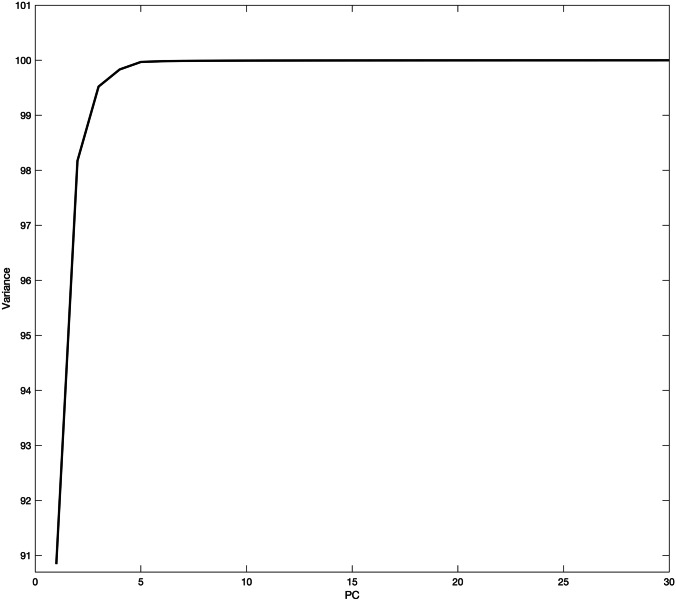
Cumulative variance of PC scores against the number of PCs

**Fig. 4:**
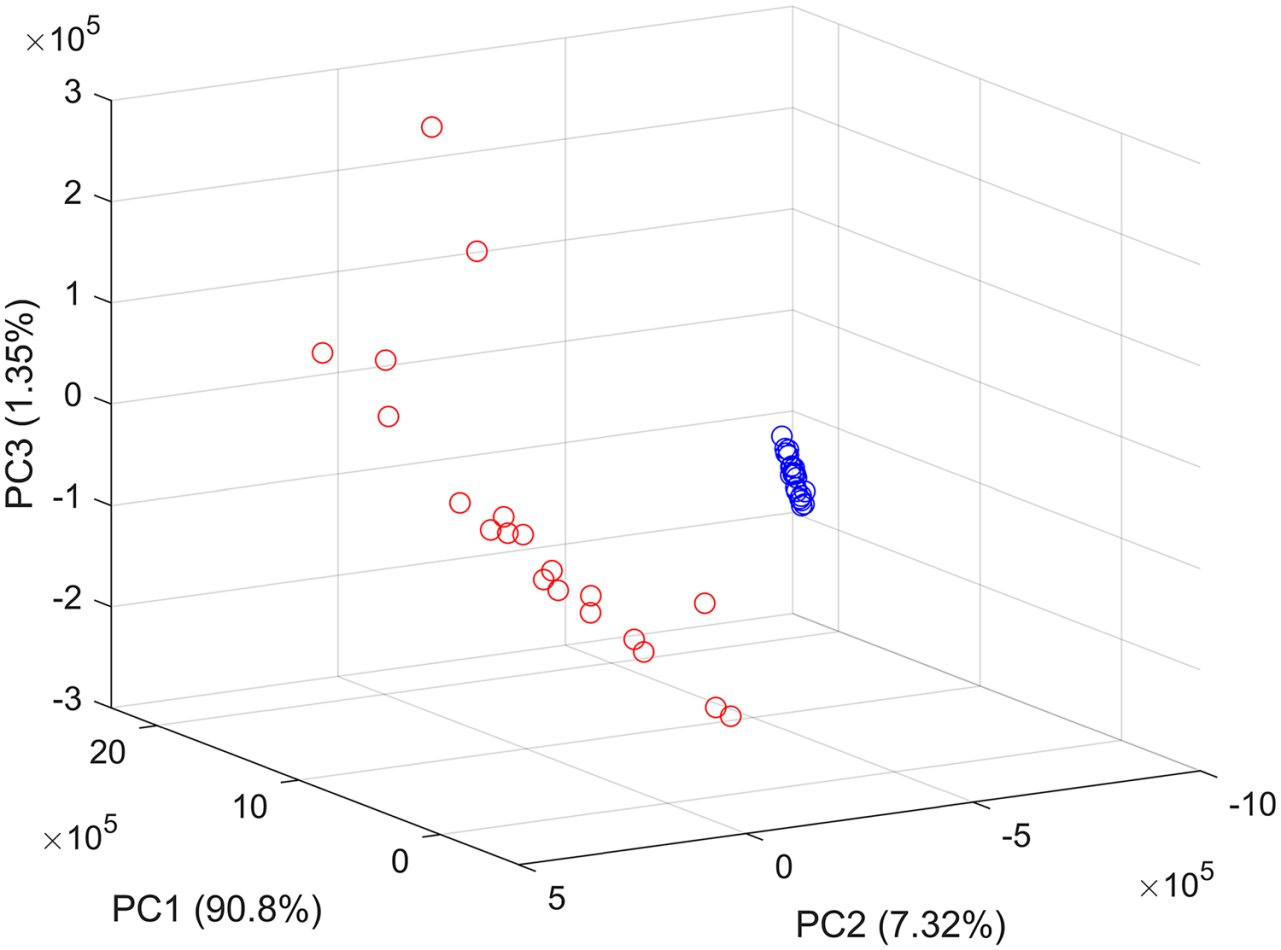
PCA scores plots of the synchronous fluorescence spectra of DNA-based vaccines (blue) and RNA-based vaccines (red)

**Fig. 5:**
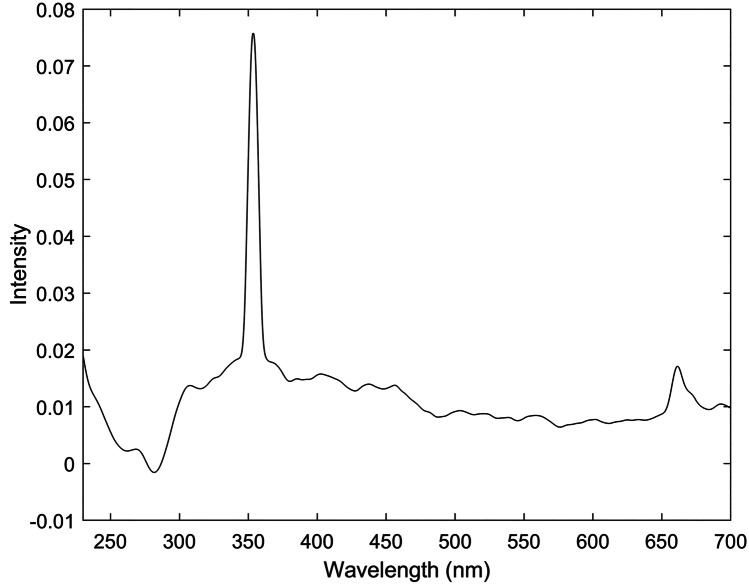
PC1 loading plots of the PCA model applied to the DNA- and RNA-based vaccines’ synchronous fluorescence spectra contributing to 90.8% of the variance among the data

When PCA was applied, the first three PCs contributed to 99.5% of the variance among the data as follow: PC1 represented 90.8%, PC2 7.32% and PC3 1.35% (Fig. 3). The PCA scores plot showed clear grouping between DNA- and RNA-based vaccines (Fig. 4). When PC loading were examined PC1 loading showed key contribution from both DNA- and mRNA-based vaccines with significant intensities around 260, 290, 354 and 660 nm respectively (Fig. 5). The same bands were seen significant at PC2 loading and that explained the model representation of the vaccines’ spectra. Where PC clusters were examined, the PC scores plot showed two distinct clusters corresponding to the DNA-based vaccines and mRNA-based vaccines respectively. Though both groups were clustered separately there was no precision in the group of mRNA-based vaccine. Hence, the scores of mRNA-based vaccines showed distances within their individual scores and that could be attributed to the variation in mRNA that attributes to individual variations in the nucleic acids and fats between the samples [40]. Hence, PCA was able to inform about variations in spectra that could not be identified by visual inspection.

#### Gaussian Clustering

**Fig. 6:**
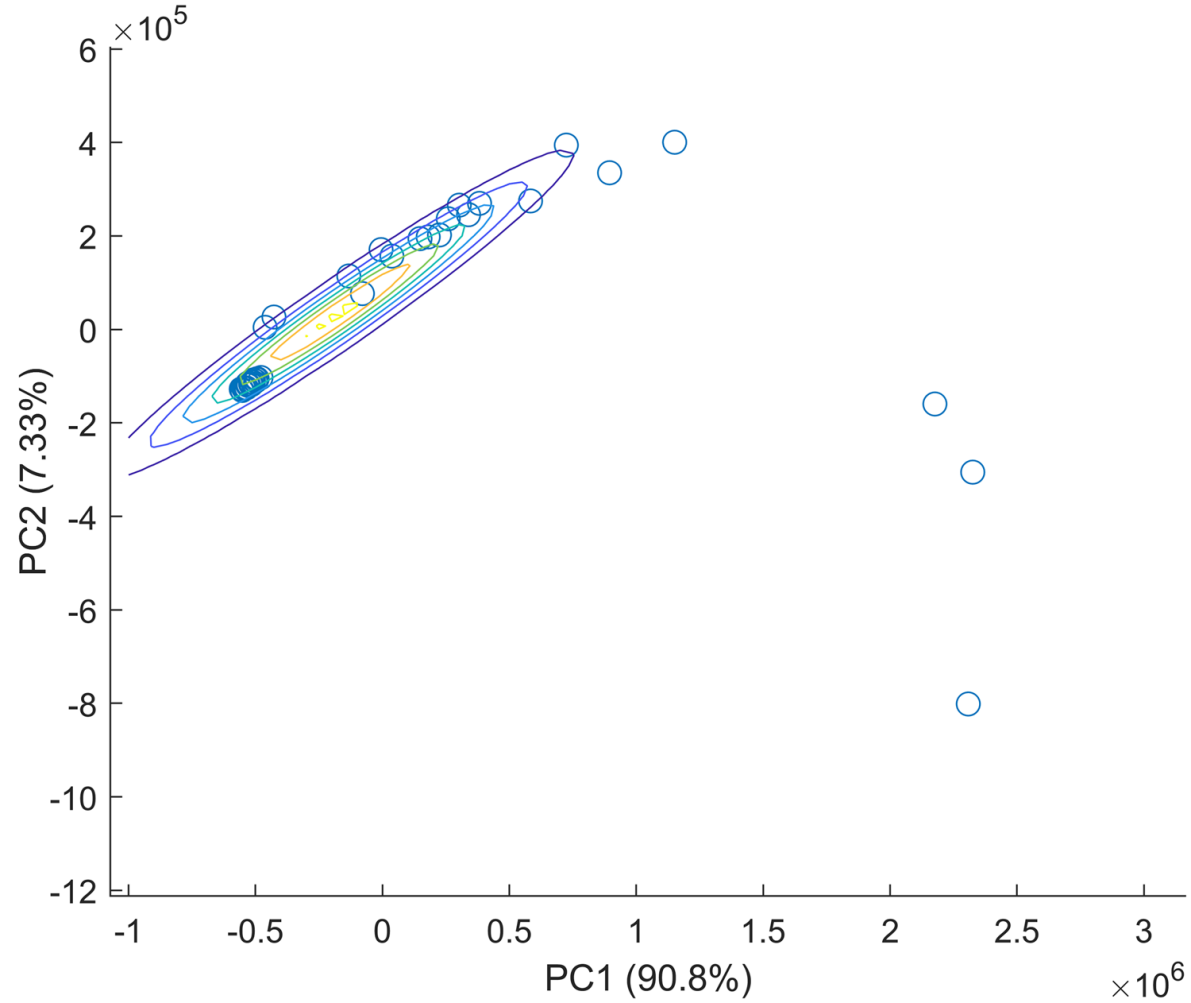
PCA-GMM clustering of the synchronous fluorescence spectra of DNA-based vaccines (blue) and RNA-based vaccines (red)

GMM clustering complemented the PCA clusters by further classifying the mRNA-based vaccines (Fig. 6). The GMM clusters showed three mRNA-based vaccines not grouped with the remaining vaccines. On the other hand, the remaining three mRNA-based vaccines were clustered within the same covariance matrix as the DNA-based vaccines. This latter finding showed that although GMM clustering could add to the information identified by PCA; it could not classify efficiently DNA- and mRNA-based vaccines.

#### Self-organising Maps

**Fig. 7:**
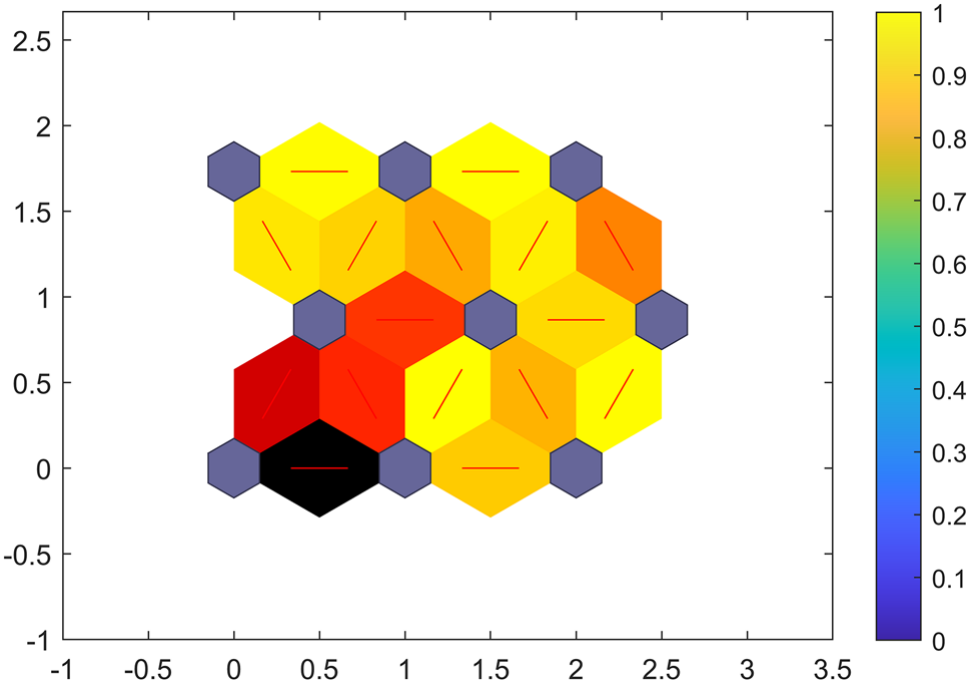
U-Matrix for the synchronous fluorescence spectra of DNA-based and RNA-based vaccines

**Fig. 8:**
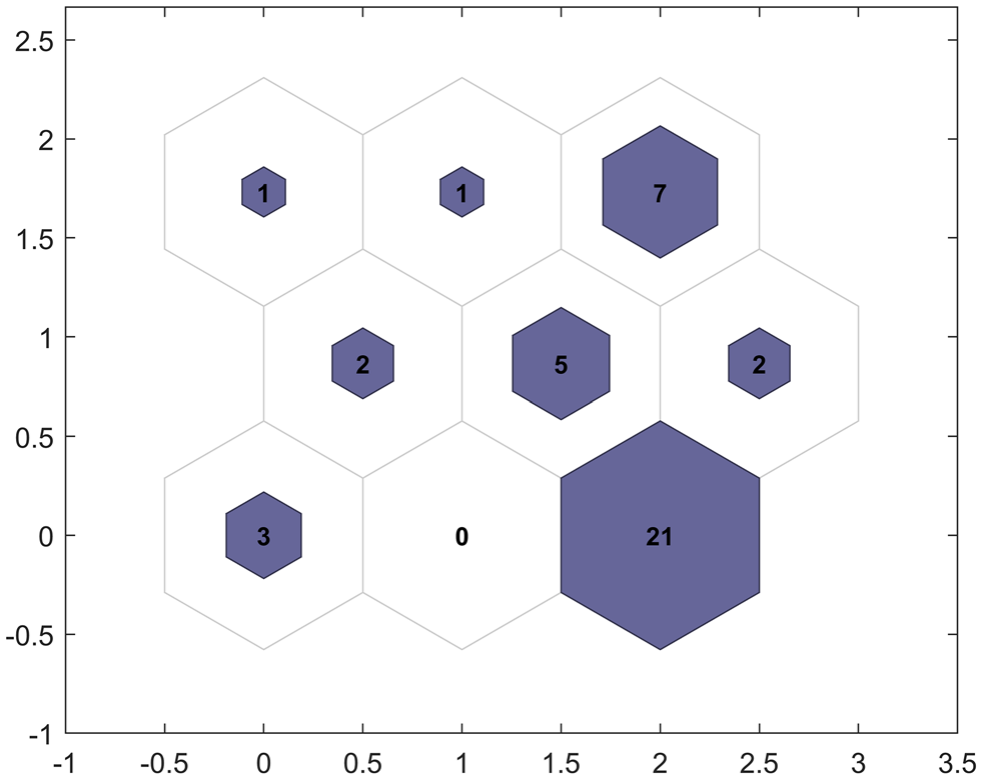
Label-Matrix for the synchronous fluorescence spectra of DNA-based and RNA-based vaccines

The U-Matrix showed the weight distances that expressed the distances between neighbouring neurons. Neurons featured in (Fig. 7) as purple hexagons and were connected to each other by the red lines (distance between neurons). Smaller distances had higher densities and larger distances had lower distances. The map size chosen was small (3 × 3) considering the three variations between vaccines related to DNA, mRNA and other additives. The algorithm grouped the vaccines into several groups and that was not dependant of the number of iterations. In this respect, the 21 DNA-based vaccines were all associated with the same group; whereas, the mRNA-based vaccines were grouped into seven groups and that corresponded to the individual variation between the vaccines (Fig. 8). Hence, SOM complemented both PCA and PCA-GMM and showed to be powerful with multivariate data without the need for dimensions reduction.

## Conclusions

The present work proposed a novel synchronous method for authentication of Covid-19 vaccines by spectral visualisation and using three classification algorithms being PCA, PCA-GMM and SOM. Spectral visualisation of DNA- and mRNA-based vaccines showed key bands corresponding to the (6–4) photoproducts of thymine and uracil respectively. In addition, classification results showed that PCA outperformed GMM in differentiating between the two main groups of DNA- and mRNA-based vaccines. At the same time, PCA indicated outliers within a specific group. On the other hand, SOM did not require any previous clustering as the case of PCA-GMM. Moreover, SOM could classify DNA-based vaccines and show the different groups of mRNA-based vaccines. Therefore, future will involve further trials with SOM by using larger sample size that was not possible in this case considering the type and accessibility of the present samples. Moreover, other spectroscopic techniques such as infrared and Raman spectroscopy may offer more information about constituents within vaccines in addition to DNA and RNA.

## Data Availability

Data will be made available upon reasonable requests from authors.
